# Naturally Occurring Bioactives as Antivirals: Emphasis on Coronavirus Infection

**DOI:** 10.3389/fphar.2021.575877

**Published:** 2021-06-29

**Authors:** Seyed Abdulmajid Ayatollahi, Javad Sharifi-Rad, Patrick Valere Tsouh Fokou, Gail B. Mahady, Hafiz Ansar Rasul Suleria, Shivani Krishna Kapuganti, Kundlik Gadhave, Rajanish Giri, Neha Garg, Rohit Sharma, Daniel Ribeiro, Célia F. Rodrigues, Željko Reiner, Yasaman Taheri, Natália Cruz-Martins

**Affiliations:** ^1^ Phytochemistry Research Center, Shahid Beheshti University of Medical Sciences, Tehran, Iran; ^2^ Department of Pharmacognosy and Biotechnology, School of Pharmacy, Shahid Beheshti University of Medical Sciences, Tehran, Iran; ^3^ H.E.J. Research Institute of Chemistry, International Center for Chemical and Biological Sciences, University of Karachi, Karachi, Pakistan; ^4^ Department of Biochemistry, Faculty of Science, University of Bamenda, Bamenda, Cameroon; ^5^ Department of Pharmacy Practice, PAHO/WHO Collaborating Centre for Traditional Medicine, College of Pharmacy, University of Illinois at Chicago, Chicago, IL, United States; ^6^ Department of Agriculture and Food Systems, University of Melbourne, Melbourne, VIC, Australia; ^7^ School of Basic Sciences, Indian Institute of Technology Mandi, Mandi, India; ^8^ Department of Medicinal Chemistry, Faculty of Ayurveda, Institute of Medical Sciences, Banaras Hindu University, Varanasi, India; ^9^ Department of Rasa Shastra and Bhaishajya Kalpana, Faculty of Ayurveda, Institute of Medical Sciences, Banaras Hindu University, Varanasi, India; ^10^ Northern Superior Health School of the Portuguese Red Cross, Oliveira de Azeméis, Portugal; ^11^ Instituto de Investigação e Formação Avançada em Ciências e Tecnologias da Saúde, Rua Central de Gandra, Gandra, Portugal; ^12^ LEPABE—Laboratory for Process Engineering, Environment, Biotechnology and Energy, Faculty of Engineering, University of Porto, Porto, Portugal; ^13^ Department of Internal Medicine, University Hospital Centre Zagreb, School of Medicine, University of Zagreb, Zagreb, Croatia; ^14^ Laboratory of Neuropsychophysiology, Faculty of Psychology and Education Sciences, University of Porto, Porto, Portugal; ^15^ Department of Biomedicine/Medicine, Faculty of Medicine, University of Porto, Porto, Portugal; ^16^ Institute for Research and Innovation in Health, University of Porto, Porto, Portugal

**Keywords:** Coronavirus, SARS-CoV-2, bioactive compounds, natural compounds, COVID-19

## Abstract

The current coronavirus disease (COVID-19) outbreak is a significant threat to human health and the worldwide economy. Coronaviruses cause a variety of diseases, such as pneumonia-like upper respiratory tract illnesses, gastroenteritis, encephalitis, multiple organ failure involving lungs and kidneys which might cause death. Since the pandemic started there have been more than 107 million COVID-19 infections caused by severe acute respiratory syndrome coronavirus 2 (SARS-CoV-2) and ∼2.4 million deaths globally. SARS-CoV-2 is easily transmitted from person-to-person and has spread quickly across all continents. With the continued increase in morbidity and mortality caused by COVID-19, and the damage to the global economy, there is an urgent need for effective prevention and treatment strategies. The advent of safe and effective vaccines has been a significant step forward in the battle against COVID-19, however treatment of the symptoms associated with the disease still requires new anti-viral and anti-inflammatory drug therapies. To this end, scientists have been investigating available natural products that may be effective against SARS-CoV-2, with some products showing promise in fighting several viral infections. Since many natural products are dietary components or are prepared as dietary supplements people tend to consider them safer than synthetic drugs. For example, Traditional Chinese Medicines have been effectively utilized to treat SARS-CoV-2 infected patients with promising results. In this review, we summarize the current knowledge of COVID-19 therapies and the therapeutic potential of medicinal plant extracts and natural compounds for the treatment of several viral infections, with special emphasis on SARS-CoV-2 infection. Realistic strategies that can be employed for the effective use of bioactive compounds for anti-SARS-CoV-2 research are also provided.

## Introduction

Coronavirus disease (COVID-19) is caused by severe acute respiratory syndrome coronavirus 2 (SARS-CoV-2) that has endangered the whole world and its pandemic is of global public health concern (Rothan and Byrareddy, 2020). Regardless of rigorous control in many countries and quarantine efforts, the spread of COVID-19 is still ongoing. Coronaviruses (CoV) were initially thought to be associated with mild respiratory illnesses in humans and not fatal, until the recent outbreak. However, the current CoV infection, SARS-CoV-2, has proven to be one of the most pathogenic and contagious viral infections, presenting clinically as severe atypical pneumonia and severe acute respiratory diseases (Perlman and Netland, 2009; Lauber et al., 2012; Coronavirus disease, 2019). Briefly, SARS-CoV-2 infection is characterized by sore throat, high fever, chills, cough, breathlessness, severe pneumonia, and death due to multi-organ failure involving kidneys and lungs often leading to death (Coronavirus disease, 2019; WHO Novel Coronavirus–China).

Currently, COVID-19 is thought to be a zoonosis, with the virus being transmitted from animals to humans, then mutating to promote human-to-human transmission (Coronavirus disease, 2019). SARS-CoV-2 is similar to SARS-CoV (also a zoonotic CoV) that first appeared in 2002 in Southern China, then spread throughout the world (Coronavirus disease, 2019; Hui and Zumla, 2019). In 2012, another novel strain of CoV emerged, also causing a SARS-like disease epidemy, although it did not develop into a pandemic. MERS (MERS-CoV, another zoonotic CoV) was endemic to the Middle East, with a particularly high fatality rate (around 35%) (Ajlan et al., 2014; Azhar et al., 2019), and was thought to be caused by contact with camels or camel-based products during the 2012–2013 outbreak (Azhar et al., 2019). Interestingly, birds and other mammals also suffer from a variety of CoV infections, which are mostly fatal, such as the infectious bronchitis virus (IBV) in poultry, transmissible gastroenteritis virus (TGEV) in pigs and bovine coronavirus (BCoV) in cattle, all associated with huge economic losses(Pyrc et al., 2008).

In terms of treatment of COVID-19 infections, the current focus has been to test already approved antiviral medications, molecules that bind to the virus, such as antibodies, siRNA, ribozymes, and many natural products, including Traditional Chinese Medicines (TCM) (Gu and Korteweg, 2007; Kumar et al., 2013). In fact the use of natural products and traditional plant-based medicines for the treatment of COVID-19 is common in many developing countries, and investigations of naturally derived bioactive substances have shown promising anti-viral effects against a number of viruses through multiple mechanisms (Suwannarach et al., 2020; Mukhtar et al., 2008). Additionally, naturally occurring bioactive substances from plants have significant anti-inflammatory, antifungal, antibacterial, and immunomodulatory effects (Suwannarach et al., 2020; Mukhtar et al., 2008). Thus, considering that natural plant derivatives are already reported to have antiviral effects, they may be good options to be explored for the research and development of novel anti-SARS-CoV-2 therapeutics and value added approaches to current therapies. The aim of this review was to provide an overview of SARS-CoV-2, and current therapies with a focus on the effects of naturally occurring medicinal plant extracts and bioactive compounds on CoVs, to outline their possible development for the prevention and treatment of SARS-CoV-2 infection.

## Coronaviruses: A Brief Overview

**FIGURE 1:**
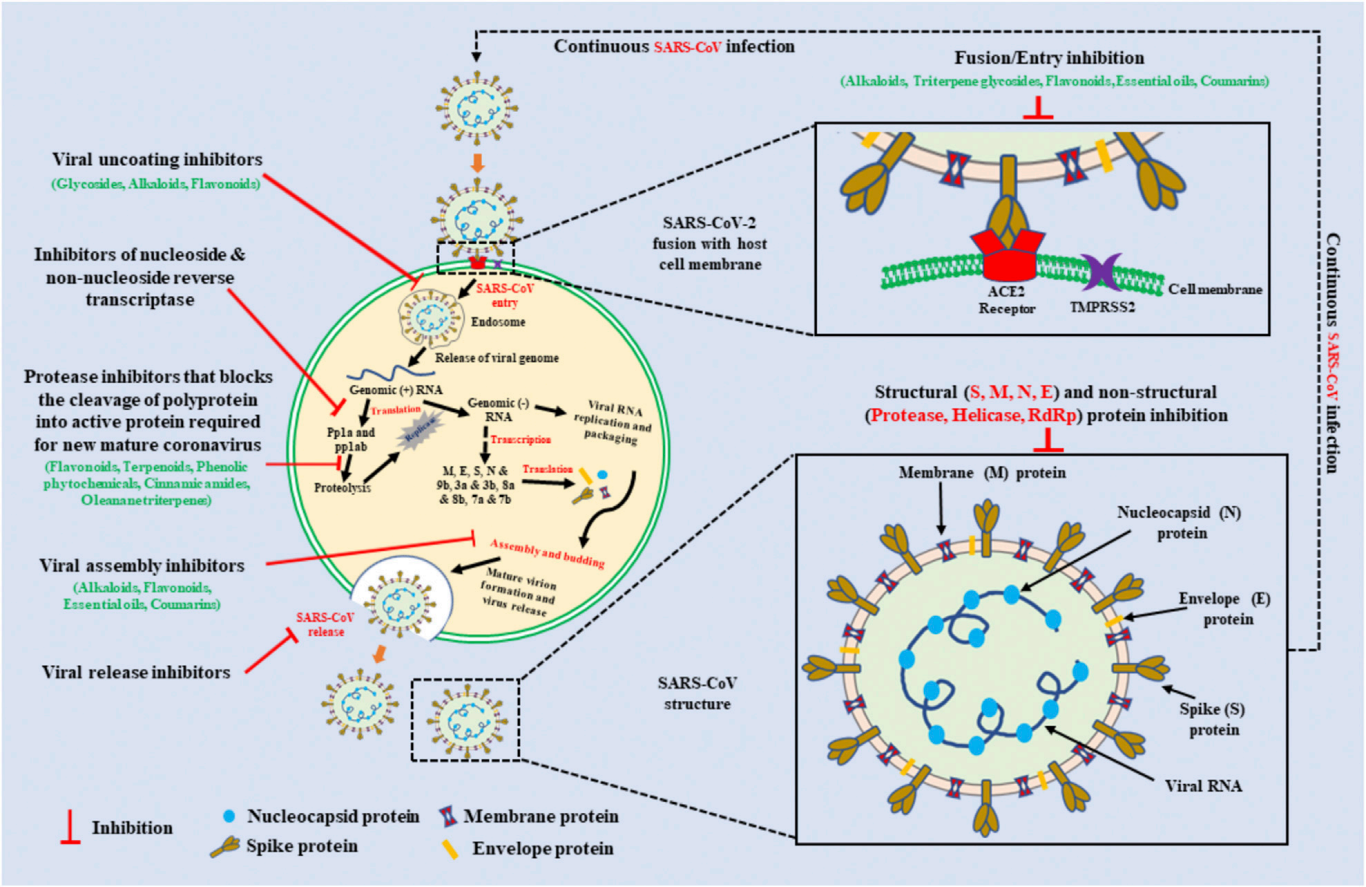
Schematic representation of a Coronavirus replication cycle, SARS-CoV virus structure, and the molecular targets for naturally occurring plant extracts and their active compounds against on viral infection. For attachment with host cells, SARS-CoV S protein use the cellular attachment factor Angiotensin-converting enzyme 2 (ACE2) and the cellular protease Transmembrane protease serine 2 (TMPRSS2) for their activation. SARS-CoV enters into host cells via endocytosis and release its RNA genome inside the cell. Further early and late protein synthesis occurs. Finally, viral assembly and then release outside the cells via exocytosis. The natural compounds that can target at different stages of viral replication may inhibit SARS-CoV infection. Inhibitors derived from plant sources such as alkaloids, glycosides, phenolic phytochemicals, essential oils, coumarins, cinnamic amides, and oleanane triterpenes may provide great treatment. Schematic diagram of SARS-CoV virus structure represents the single stranded positive-sense viral RNA, Spike (S), Nucleocapsid (N), Membrane (M), and Envelope (E) protein. Bioactive compounds that can inhibit structural and non-structural proteins of coronaviruses can be used with inhibitors of viral replication cycle.

Coronaviruses (CoVs) are characteristically enveloped, with a positive-sense single-stranded RNA genome, a lipid membrane originating from the host cell, and club-like spikes on their surfaces (Figure 1). (Song et al., 2004) Proteins protruding from viral membrane gives the virus its characteristic halo-like appearance, which is the reason for the name “corona” (Ludwig and Zarbock, 2020). CoVs belong to the order Nidovirales, that includes the families: arteriviridae, Coronoviridae, Mesoviridae and the Roniviridae. The Coronoviridae is comprised of the Coronavirinae and Torovirinae subfamilies. Coronavirinae subfamily is further divided into four genera: alpha (α), beta (β), gamma (γ), and delta (δ) CoVs (Cui et al., 2019). Among these, α- and β-CoV can infect mammals, whereas γ- and δ-CoV mainly infect birds, however CoVs are found in humans and several animal species (Ludwig and Zarbock, 2020), and have high mutation rates thereby enabling CoVs to infect different species (Duffy et al., 2008). Most recently, a novel member of the human CoV emerged and is now formally named as SARS-CoV-2 (severe acute respiratory syndrome coronavirus 2) (Ludwig and Zarbock, 2020). This unique strain of CoV was previously reported in humans, and is able to cause disease in humans, masked palm civets, mice, dogs, cats, camels, pigs, chickens, and bats. SARS-CoV-2 has been observed to cause severe respiratory and gastrointestinal sickness in animals and humans and may be transmitted through aerosols and direct/indirect contact.

Some specific structural proteins have been identified in the virus that play significant roles in its pathogenesis and development of severe infection. These protein molecules are encoded by the SARS-CoV genome and have been identified as possible targets for new anti-CoV agents. These proteins include: the spike (S) protein that allows for the entry of the virus into cells; the SARS-CoV chymotrypsin 3C-like protease (3CL^pro^) that is required for the viral life cycle; the ntpase/helicase, RNA-dependent RNA polymerase (RDRP); the membrane (M) protein that is required for viral budding; the envelope (E) protein that plays a role in CoV assembly; and the nucleocapsid (N) phosphoprotein that is related to viral RNA inside the virion and possibly other viral protein-mediated processes. By binding to and interacting with these critical proteins, drugs and natural compounds may prevent infection or alter viral replication and spread. Thus, a better understanding of these proteins, and other important components of the SARS-CoV, may increase investigations of novel anti-SARS-CoV agents using a targeted approach against these specific proteins.

### Characteristics of Coronavirus Proteins

CoVs have a spherical structure with ∼125 nm diameter (Fehr and Perlman, 2015). The genome consists of a positive single-stranded RNA with a 5′ cap and a 3′ poly-A tail (Figure 1). Around 20 kb of the genome at the 5’ end consists of a replicase gene, which encodes about 16 non-structural proteins (Snijder et al., 2016). The structural spike (S), nucleocapsid (N), envelope (E), and membrane (M) and the accessory proteins are encoded by the rest of the genome (Nga et al., 2011). The replicase gene encodes for open reading frames (ORFs), rep1a and rep1b, which express respectively the polyproteins pp1a and pp1ab. Translation of both polyproteins from the same ORF is mediated by a slippery sequence and ribosomal frameshifting caused by an RNA pseudoknot (Brierley et al., 1989; Baranov et al., 2005). The nsps 1–11 occur in pp1a whereas nsps 1–16 occur in pp1ab (Ziebuhr et al., 2000).

The spike glycoprotein (∼150 kDa) is a type-1 transmembrane protein, with around 20–25 N-glycosylation sites and is present as a homotrimer on viral surface. The protein has two domains, an N-terminal S1 domain, and a C-terminal S2 domain. The S1 domain has the receptor binding sites and the S2 domain contains two heptad repeats that mediate viral fusion with the host cell. The S protein attaches to different receptors on the host cell based on the type of coronavirus. For example, α-CoV interact with aminopeptidase N, whereas SARS-CoV and HCoV-NL63 interact with angiotensin-converting enzyme 2 (ACE2) on the host cell surface. Other known receptors include CEACAM1 and dipeptidyl-peptidase 4, respectively, used by MHV and MERS-CoV to enter the host cells (Kubo et al., 1994; Bosch et al., 2003; Belouzard et al., 2009).

The membrane glycoprotein (∼30 kDa) is responsible for giving the viral shape. It is a dimer which promotes the membrane curvature and binds to nucleocapsid-RNA complex during virus packaging. The M protein has three domains: an N-terminal ectodomain, followed by three transmembrane domains, and a C-terminal endodomain. The ectodomain is either O- or N-linked, glycosylated depending on the type of the virus and is susceptible to proteolytic cleaving (Nal et al., 2005; Neuman et al., 2011).

The envelope protein (∼8–12 kDa) is present at limited quantities in the virus. The protein sequence varies among distinct viruses, although structurally similar. It is a transmembrane protein with ion channel activity. Its main role is in the assembly and release of viral particles (Nieto-Torres et al., 2014).

The N protein (∼45–50 kDa) is the protein component of the helical nucleocapsid. Both the N- and C-terminal domains of the protein bind to RNA in a phosphorylation-dependent, beads-on-string manner (Chang et al., 2006). The N protein interacts with transcription regulating sequences, regions of 3’ UTR on viral RNA, and genomic RNA packaging signals (Cologna et al., 2000; Chen et al., 2005). Nuclear localization of N protein in some viruses has been detected, although its role in viral replication is not yet known. N protein helps in packaging the viral RNA into the virus, besides to interacts with NS3 and M protein (Sturman et al., 1980; McBride et al., 2014).

A subset of β-CoVs have an additional hemagglutinin-esterase structural protein. There is some speculation that this protein may enhance viral entry and spread through interactions with sialic acids on the surface glycoproteins, acting as hemagglutinin and acetyl-esterase (Klausegger et al., 1999). Apart from the replicase and structural genes, CoVs have additional ORFs interspersed between and/or overlapping with these genes that encode for accessory proteins. CoVs can have one to nine accessory proteins (ORF3a, ORF3b, ORF6, ORF7a, ORF7b, ORF8, ORF9, ORF10, and ORF14) depending on the virus type (Nga et al., 2011). Most are nonstructural proteins, but sometimes they can be a part of the virus structure, for instance the SARS-CoV 3a protein (Fischer et al., 1997). In the most studied animal CoV, murine hepatitis virus, accessory proteins have been found unnecessary for viral replication in tissue culture, although they seemed to enhance the *in vivo* virulence (Narayanan et al., 2008).

The non-structural CoVs proteins play distinct roles in virus-mediated infection. For example, Nsp1 helps in blocking host cell translation and immune response, conferring a favorable environment for virus propagation (Huang et al., 2011; Tanaka et al., 2012). Nsp3 is a papain-like protease that cleaves the nsp1/nsp2, nsp2/nsp3, and nsp3/nsp4 boundaries (Ziebuhr et al., 2000). It also prevents host cell degradation, which is required for proper host proteome functioning. Nsp4 and Nsp6 are transmembrane proteins that may act as a basis for the double membraned vesicles where virus replication and assembly take place (Oostra et al., 2008; Gadlage et al., 2010). Nsp5, also known as Mpro, is a serine-like protease that catalyzes the remaining 11 cleavage events of the replicase gene product (Ziebuhr and Siddell, 1999; Ziebuhr et al., 2000; Ziebuhr, 2005). Nsps7 and eight act as processivity clamps for the polymerase, Nsp12 (Zhai et al., 2005). Nsp10 is a cofactor for Nsp16, which protects viral RNA from MDA5 recognition and viral RNA from host antiviral mechanisms (Bouvet et al., 2010; Decroly et al., 2011). Nsp12 is a RNA-dependent RNA polymerase. Nsp13 is a RNA helicase with a 5′-triphosphatase activity (Ivanov et al., 2004; Ivanov and Ziebuhr, 2004; Minskaia et al., 2006), and Nsp14 is a methyltransferase (mtase) that adds 5′ cap to viral RNA, also having a 3′-5′ exonuclease activity required for viral genome proofreading (Chen et al., 2009). Nsp15 is an endoribonuclease that cleaves extra viral RNA as a defensive measure from host attacks. The functions of other Nsps are not yet clear.

### Mechanisms of Infection and Targeted Tissues

Coronaviruses are highly contagious, and may be spread by inhalation or ingestion of virus-containing droplets, leading to clinical symptoms, such as coughing and sneezing among others (Boopathi et al., 2020). Viral N protein allows the virus to hijack human cell mechanisms to create viral factories (Boopathi et al., 2020). For penetration into the host cell, CoVs depend on envelope fusion with the host cell membrane, and the S protein facilitates the CoVs entry into host cells by binding with host hACE2 receptors (Belouzard et al., 2012; Ou et al., 2020). After the interaction with the hACE2 receptors, the S protein undergoes acid-dependent cleavage by a host protease at two sites. The first cleavage separates the receptor-binding site and the fusion domain on the S protein, while the second cleavage exposes the fusion peptide S2’, that mediates viral fusion with host membranes. Translation of the replicase gene from the viral genome occurs and both genomic and sub-genomic viral RNAs are synthesized by negative-strand intermediates. Sub-genomic RNAs code for structural and accessory proteins, which are translated and inserted into the endoplasmic reticulum (ER) and move along the ER-Golgi intermediate compartment where virus assembly takes place (Fehr and Perlman, 2015). The N protein with bound viral RNA forms budding structures at the compartment membrane and M protein incorporate other proteins into the virus structure by interacting with them and the nucleocapsid. Finally, the assembled virus particles are released from the host cell by exocytosis (Tooze et al., 1984; Krijnse-Locker et al., 1994).

In humans, CoV infections were linked only to mild respiratory illnesses until the advent of the 2002/2003 SARS-CoV outbreak, then the 2012/2013 MERS-CoV outbreak, and finally the 2019/2020 SARS-CoV-2 pandemic. These outbreaks were associated with severe pneumonia-like respiratory illness and death due to multi-organ failure. Human CoVs primarily infect immunocompromized individuals, such as the elderly, and patients suffering from other chronic diseases including diabetes, hypertension, obesity and heart disease, with most fatalities reported in patients over the age of 50 (Pene et al., 2003; Gorse et al., 2009). CoVs start by infecting the lung epithelium, then enter macrophages and dendritic cells producing significant proinflammatory cytokines and chemokine secretion called “cytokine storm”. This cytokine storm, led by interleukin (IL)-6, is a severe response to viral infection, and initiate molecular events that are the basis of multi-organ failure and death associated with COVID-19 infections (Gu and Korteweg, 2007; Van Der Hoek et al., 2005). CoVs further use host systems for their propagation, and by inhibiting host translation, CoVs convert host translation mechanisms to viral translation (Fung et al., 2016). Viral protein synthesis induces ER stress, which in turn induces unfolded protein response (UPR) that inhibits host translation by protein kinase RNA-like ER kinase (PERK)-induced phosphorylation of the translation initiating factor eIF2α. Also, Nsp1 inhibits the initial steps of translation, such as the conversion of 48 initiation complex into 80s complex, which could be seen in SARS-CoV (Lokugamage et al., 2012). Moreover, CoVs have developed mechanisms to escape the host innate immune system. MDA-5 and toll-like receptors (TLRs) are host factors that detect viral RNA and proteins (Bruns and Horvath, 2014; Totura et al., 2015). Activation of such “sensors” leads to the activation of interferon (IFN) signaling and nuclear factor-κB (NF-κB) pathway and induces IFN-stimulated genes by the Janus kinase (JAK)-signal transducer and activator of transcription (STAT) (JAK-STAT) signaling cascade (Schoggins and Rice, 2011). However, different CoVs have evolved different strategies of immune system evasion. As an example, the Nsp1 protein of SARS-CoV and the N protein of MHV suppress IFN signaling, and accessory proteins, such as ORF3b and ORF6 of SARS-CoV, ORF4a, 4b, and five of MERS-CoV have also been reported to inhibit IFN signaling (Kopecky-Bromberg et al., 2007; Siu et al., 2014). Subsequently, the Nsp3 overexpression suppresses IFN signaling that causes interference in both host immune signaling factors processing and function (Matthews et al., 2014; Li et al., 2016). Moreover, Nsp16 mediates 2′-O methylation of viral RNAs masking it from MDA5 (Menachery et al., 2014). Thus, taken together, such immune suppression strategies help to enhance the pathogenicity of CoV infections.

Minor changes in the S protein have been observed in CoV, that may alter the infectivity of the virus in different hosts. For example, hSARS-CoV can infect both palm civets and humans, whereas palm civet SARS-CoV cannot infect humans. For hSARS-CoV, two-point mutations in the S protein were identified, that allow the S protein to bind to the hACE-2 receptor (Li et al., 2005). Similarly, transmissible gastroenteritis coronavirus (TGEV) and porcine respiratory coronavirus (PRCoV), both bind to porcine aminopeptidase N (APN). Due to a deletion in the N-terminal domain of the S protein, TGEV infects the epithelial cells of both respiratory tract and small intestine, while PRCoV can only infect the pulmonary epithelium (Rasschaert et al., 1990; Schultze et al., 1996). Some CoVs also interact with C-type lectins on host cell surfaces. For example, DC-SIGN is a receptor on macrophages and dendritic cells, while L-SIGN is a receptor in liver and lung endothelial cells. Such receptors usually recognize and bind glycosylated viral antigens. In this way, SARS-COV, human CoV 229E (HCoV-229E), infectious bronchitis virus (IBV), and feline CoV (FCoV) can infect these cells by interacting with these receptors by their highly glycosylated S proteins (Jeffers et al., 2004; Jeffers et al., 2006). For example, some IBV strains cause urinogenital and reproductive tract infection in chicken, but also respiratory disease (Perlman and Netland, 2009), while mouse hepatitis virus (or murine CoV, MHV), strain A59 may cause hepatic and enteric infections, and strain JHMV causes neurological disorders similar to multiple sclerosis in mice (Lampert et al., 1973; Houtman and Fleming, 1996). Overall, CoVs have a wide range of tissue tropism from lungs, gut, liver, kidneys, reproductive tract to nervous system.

## Animal and Human Infections by Coronavirus

### Animal Infections by Coronavirus

There are some indications that SARS-CoV-2 is a zoonosis, a disease originating from animals and transmitted to humans. Overall, CoVs are known to cause multiple health implications, including enteritis, hepatitis, respiratory illnesses, encephalitis, demyelinating disease, urinary and reproductive tract infections, with symptoms such as diarrhea, cough, wasting, decreased milk or egg production being also present, in both mammals and birds. Below, are briefly described some examples:

**-** Avian infectious bronchitis virus, first discovered in the 1930s, and often causes fatal respiratory illnesses in young chickens and a decrease in eggs production. It spreads by oral-fecal route and air. Approximately eight viral serotypes have been characterized. The primary target is trachea, although it can also infect the bronchia, kidneys, and reproductive tract, including ovaries and oviducts. Antiviral antibodies can be detected three days after the infection (Wege et al., 1982; Jackwood, 2012).
**-** Turkey CoV was first detected in the 1950s. Infection triggers transmissible enteritis with symptoms like diarrhea, weight loss, and depression in turkeys with low mortality rates. Its primary target is the gut, leading to loss of microvilli, hemorrhage, and loss of goblet cells in the small intestine. These symptoms can be found 1 day after the infection (Wege et al., 1982).
**-** Bovine CoV is transmitted by the oral route and causes bovine viral diarrhea in young calves. It can also affect different ruminant, such as camels, elks, and deer. The primary target is the intestinal absorptive epithelium, with consequent extensive loss of water and ions. These symptoms can be seen within 20–30 h post-infection. It has been suggested that offspring-transmitted maternal antibodies can induce a certain degree of protection against the infection (Wege et al., 1982; Saif, 2010).
**-** Canine CoV was first detected in military dogs in the 1970s, with symptoms including mild gastroenteritis (vomiting and diarrhea). It is transmitted orally and the symptoms are observed within a week after the infection. Target tissues include small and large intestine and lymph nodes (Wege et al., 1982).
**-** Hemagglutinating encephalomyelitis virus causes vomiting and neurological symptoms in pigs, and often leads to death. It is transmitted by the oronasal route, primarily infecting the respiratory tract, tonsils, and then the small intestine from where it moves to peripheral ganglia through nerves. Symptoms appear 4 days after the infection. Neuronal tissue infection causes small intestine paralysis, leading to starvation and even death (Wege et al., 1982).
**-** Transmissible gastroenteritis virus causes vomiting and diarrhea in pigs, often causing death. It is transmitted orally and it infects the absorptive small intestine cells, despite respiratory disease can also occurs. Antibodies can be transferred from mother to suckling pigs.
**-** Porcine epidemic diarrhea virus causes severe gastroenteritis in piglets and is associated with high mortality.
- Porcine hemagglutinating encephalomyelitis virus causes vomiting, decay, encephalitis and enteritis in pigs. Different vaccines have been developed for transmissible gastroenteritis virus (TEGV) and porcine epidemic diarrhea virus (PEDV) (Perlman and Netland, 2009; Langel et al., 2016; Gerdts and Zakhartchouk, 2017; Pascual-Iglesias et al., 2019; Xue et al., 2019).
**-** Rat CoV is transmitted nasally and causes fatal respiratory disease in rats. The primary targets of infection are nasal epithelium and lungs. Another strain, the sialodacryoadenitis virus, infects the salivary and lacrimal glands. Infection spreads through the respiratory tract to the lymph nodes and eventually affects the salivary glands causing rhinitis and necrosis of the gland duct epithelium (Wege et al., 1982).
**-** Feline infectious peritonitis virus (FIPV) affects wild and domestic cats. It infects a wide range of tissues, such as trachea, intestine, liver, kidneys, and reticuloendothelial system. It causes loss of appetite, depression, fever, neurological symptoms, pleuritis, peritonitis, fibrin deposition in abdominal organs, proteinuria, and anemia. Data have shown that a high level of antibodies cannot prevent the infection, suggesting that this could be an immunopathological disease. Live, attenuated FIPV that contains a deletion of the group-specific gene, the 3abc cluster, was reported to protect cats from the lethal homologous challenge (Haijema et al., 2004).
**-** Murine hepatitis virus (MHV) strain-JHM-was first isolated in the 1940s from spontaneously paralyzed mice. It can be transmitted by urine, feces, intrauterine, and by respiratory route. It is responsible for diseases, like hepatitis, encephalomyelitis, and enteritis. JHM is especially neurotropic, targeting oligodendritic cells, and triggering a demyelinating disease; plaques can be found in the white matter of the central nervous system (CNS). Other strains of MHV, such as MHV-2, MHV-3, and MHV-A59 often cause fatal disease by destroying the parenchymal and Kupffer cells in the liver (Gombold et al., 1995).

### Human Infections by Coronavirus

CoVs were initially thought to cause mild respiratory infections in humans until the 2002–2003 SARS-CoV outbreak, 2012–2013 MERS-CoV outbreak, and the current 2019–2020 SARS-CoV-2 pandemic, which has infected >107 million people and caused ∼2.3 million deaths globally, but numbers are still rising. The first human CoVs were discovered in the 1960s by studying fluids from people with respiratory infections. The first detected hCoV was named B814, isolated from a boy suffering from a common cold. Later, hCoV-229E (α-CoVs) was isolated together with five other strains, and showed similarity to B814, IBV, and MHV. The infection was similar to the common cold, with symptoms such as sneezing, sore throat, headache, and runny nose. The other strains, OCH38 and OCH43 (β-CoV, lineage A) caused symptoms similar to 229E. NL63 (α-CoV) was identified in 2004 in two separate cases, one from a 7-month-old child suffering from febrile bronchiolitis, fever, conjunctivitis, coryza, and the other from an 8-month-old child with pneumonia. NL63 also causes acute laryngotracheitis. Finally, HKU1 was isolated from a 71-year-old man suffering from pneumonia, with infection symptoms being similar to common cold. These viruses cause 15–30% of the commonly occurring respiratory tract diseases. There is no direct antiviral therapy for these viruses, they naturally end their course and are most often not fatal (Su et al., 2016; Cui et al., 2019).

However, in 2002, during the SARS outbreak in Guangdong province, China, 8,273 cases were reported with 775 deaths. Interestingly, SARS-CoV (β-CoVs lineage B)-like viruses were identified in palm civets and raccoon dogs, commonly found in game-animal markets in China, suggesting that they may serve as intermediate virus reservoirs. Later, the identification of two novel bat CoVs that were more similar to SARS-CoV than any other virus showed horseshoe bats to be the natural SARS-CoV reservoirs, given that they use ACE2 receptors to infect the host. SARS-CoV-infected patients have symptoms such as myalgia and diarrhea, and other typical common cold symptoms. The virus mainly affected the respiratory and gastrointestinal tracts, liver, kidney, and brain. High pro-inflammatory cytokines and chemokines levels were also detected in infected patients, thus suggesting that deaths may result of immunopathological complications (Wang and Eaton, 2007).

In 2012, MERS-CoV (β-CoV lineage c) was identified from a 60-year-old person in Saudi Arabia who died from severe respiratory disease. Between 2012 and 2014, 855 cases with 333 deaths were reported. Symptoms begin with a sore throat, fever, cough, myalgia, and progress to severe pneumonia, septic shock, and death from kidney failure. Anti-MERS-CoV antibodies and similar MERS-CoV particles were detected in dromedary camels that live in close association with humans in Saudi Arabia. Moreover, studies have shown that a bat CoVs HKU4 is phylogenetically similar to MERS-COV, where it uses DPP4 as a receptor to infect hosts (e.g. bats, humans, camels, horses and rabbits) (Memish et al., 2013).

Finally, the current COVID-19 caused by SARS-CoV-2 (Situation reports) was first detected in a wet market in Wuhan, China. Characteristically, it is very similar to bat CoVs and similar viral particles have been detected in pangolin, with such animals being sold in China’s wet markets for food purposes, and as components of TCM. Such findings raised speculations that the virus may have originated from bats, while the pangolins may have acted as intermediate hosts. Clinically, it is a rapidly spreading infection and it is transmitted from person-to-person (Zhang et al., 2020), with infected individuals presenting typical symptoms of pneumonia, sore throat, fever, chills and shortness of breath. Death occurs as a consequence of multi-organ failure, mostly involving lungs and kidneys.

## COVID-19 Infection Prevention and Therapeutic Interventions: A General Overview

Early efforts to prevent the spread of SARS-CoV-2 included early diagnosis, the isolation of infected people, frequent hand washing, wearing of masks and maintaining physical distancing have continued in most countries. Thankfully the development of vaccines for COVID-19 has been swift, and it is estimated that there are ∼66 vaccine candidates being clinically developed, with as many as 170 in pre-clinical development (Belete, 2021; Funk et al., 2020; World Health Organization; Centers for disease control, 2021; Food and Drug Administration). At least ten vaccines have been approved by at least one national regulatory authority for public use: two mRNA vaccines (Pfizer/BioNTech and Moderna vaccines), four inactivated virus vaccines: BBIBP-CorV (Beijing Institute of Biological Products/Sinopharm, China), WIBP (Wuhan Institute of Biological Products, China), and CoronaVac (Sinopharm, China), BBV152 (Bharat Biotech, India), three non-replicating viral vector vaccines: Sputnik V (Gamaleya Research Institute, Russia), the AstraZeneca/Oxford vaccine (Oxford, United Kingdom), and Ad5-nCoV (CanSino Biologics, China), and one peptide vaccine EpiVacCorona (State Research Center of Virology and Biotechnology-VECTOR, Russia) (Table 1).

**TABLE 1:** Overview of the ten vaccines that have been approved by one or more regulatory agencies worldwide.

Vaccine	Type	Company	#Shots	Efficacy %	Storage
BNT162	mRNA	Pfizer/BioNTech	2	95	−80
mRNA-1273	mRNA	Moderna	2	95	−80
BBIBP-CorV	Inactivated virus	Beijing institute of biological products/sinopharm	2	79–86	2–8
WIBP	Inactivated virus	Wuhan institute of biological products	NS	NS	NS
CoronaVac	Inactivated virus	Sinopharm	2	50.4–91.25	
BBV152	Inactivated virus	Bharat biotech/Indian medical research council	2	60–70	2–8
Sputnik V	Viral vector	Gamaleya	2	91.6	2–8
ADZ1222	Viral vector	AstraZeneca/Oxford	2	63	2–8
Ad5-nCoV	Viral vector	CanSino biologics	2	92.5	2–8
EpiVacCorona	Peptide	VECTOR	NS	NS	NS

In the United States and other countries, two mRNA vaccines have been authorized for general use to prevent COVID-19: the Pfizer-BioNTech COVID-19 (also approved by the World Health Organization, WHO) and Moderna COVID-19 vaccines (FDA). Both of these vaccines require two shots (Pfizer- 21 days apart and Moderna- 28 days a part) but are 95% effective (FDA, Table 1). In addition, three new vaccines are currently in phase three clinical trials including: the AstraZeneca-Oxford’s COVID-19 vaccine (now approved by the WHO), the Janssen COVID-19 vaccine, and the Novavax COVID-19 vaccine (Funk et al., 2020; Belete, 2021; World Health Organization; Centers for disease control, 2021; Food and Drug Administration). In China, India, and Russia, approved COVID-19 vaccines include Sputnik V (Gamelaya, Russia), BBV152 (Bharat Biotech, India) and CoronaVac (Sinovac, China). Information from clinical trials suggest that CoronaVac is anywhere from 50.4 to 91.25% effective depending on the country sponsoring the study (Funk et al., 2020; Belete, 2021; World Health Organization; Centers for disease control, 2021; Food and Drug Administration). CoronaVac is an inactivated virus vaccine that uses traditional technology similar to BBIBP-CorV and BBV152, other inactivated-virus vaccines for COVID-19 in phase III trials. CoronaVac does not need to be frozen, and both the vaccine and raw material for formulating the new doses could be transported and refrigerated at 2–8°C (36–46°F), temperatures at which flu vaccines are kept (Table 1). Reportedly, CoronaVac may be stable for up to three years in storage, that potentially offers significant advantages in vaccine distribution to regions where cold storage is an issue. The Sputnik V vaccine was developed by the Gamaleya Research Institute of Epidemiology and Microbiology and is an adenovirus viral vector vaccine (Table 1). Analysis of the clinical trial published in The Lancet, suggests that this vaccine was 91.6% efficacy without unusual side effects (Logunov et al., 2020; Logunov et al.,2021).

Interestingly, the first two FDA (United States) approved vaccines from Pfizer and Moderna were novel mRNA vaccines that differ from conventional vaccines which trigger an immune response by injecting weakened or attenuated viruses into the body. The mRNA vaccines induce the body to produce the “S” or spike protein that triggers the immune response to produce antibodies against the spike protein, thereby protecting patients from severe infections even if they become infected with SARS-CoV-2 (Funk et al., 2020; Belete, 2021; World Health Organization; Centers for disease control, 2021; Food and Drug Administration). Unlike conventional vaccines, the mRNA vaccines do not use live or even attenuated virus that causes COVID-19 thus, patients cannot get COVID-19 from the vaccine. In addition, mRNA vaccines do not impact DNA, as mRNA does not enter the cell nucleus. A major disadvantage of this type of vaccine is that it requires storage at −20°C (Moderna) and −70°C (Pfizer). Interestingly, the AstraZeneca-Oxford vaccine is made from a genetically engineered virus that causes the common cold with the intention that it will train the immune system to respond to SARS-CoV-2 infections (Table 1). It is ∼66% effective and can be stored in a fridge. The vaccines that only require refrigeration may be easier to distribute, and thus a lot more useful to developing countries that may not be able to store large amounts of vaccine at low temperatures. Many other vaccines are under development and some are in clinical trials, it is beyond the scope of this review to discuss all of the vaccine candidates, and some excellent reviews have already been published (Funk et al., 2020; Belete, 2021).

While vaccines are critical for the fight against COVID-19, they are not 100% effective, and it will take an extended period of time to vaccinate the global population, thus the development of interventional drugs to manage symptoms, reduce viral load, reduce cytokine storm, inflammation and the other symptoms of COVID-19 that cause significant morbidity and mortality should be a significant focus of scientific and medical research.

Currently, only one anti-viral drug, remdesivir, has been approved by the U.S. FDA to treat COVID-19 in adults and children over the age of 12 years of age (Food and Drug Administration). Other anti-viral drugs, such as favipiravir and merimepodib are also currently being tested. The use of the corticosteroid dexamethasone (DEXA) to reduce inflammation and treat or prevent organ dysfunction and lung injury from COVID-19 is also recommended (Food and Drug Administration). Reports have suggested that DEXA use may reduce the risk of COVID-19 related death by as much as 30% for patients on ventilators and by ∼ 20% for COVID-19 patients who require supplemental oxygen (Food and Drug Administration). In addition, the FDA has granted an emergency use authorization for baricitinib (Olumiant), a drug normally used for arthritis to treat COVID-19 in specific cases (Food and Drug Administration). Baricitinib has both anti-inflammatory and anti-viral activities and appears to reduce inflammation due to COVID-19. This opens the door for the research and development of other drugs with similar activities, including natural products that have potent anti-inflammatory and anti-viral effects. Other treatments include immune-based therapies including convalescent plasma. The FDA has also granted emergency use authorization for convalescent plasma therapy to treat COVID-19 (Food and Drug Administration). Convalescent plasma is obtained from blood donated by patients that have recovered from COVID-19 and is given by intravenous administration to other COVID-19 patients. Anti-malaria drugs, such as hydroxychloroquine and chloroquine were at one point thought to potentially be useful, however they since have been found to have death rate twice as high as patients who have not received it. Thus, it is clear that other drugs with efficacy against COVID-19 are urgently needed, and natural products can offer a value-added approach to treatment.

Since the previous SARS and MERS outbreaks, researchers have actively started looking for other investigational new drugs for the treatment and prevention strategies CoV infections. A number of small molecules and natural products are in pre-clinical tests, with studies focus being mainly viral proteins, including proteases, polymerase and the entry protein (S protein) as novel antiviral targets, since these proteins mediate the most important functions of the virus life cycles, as described above. For example, lopinavir, a protease inhibitor used to treat human immunodeficiency virus (HIV), is reported to have *in vitro* and *in vivo* activities against SARS and MERS (Chen et al., 2004; Chong et al., 2015). Nucleoside analogs are widely used for several viral diseases, such as HIV and flaviviruses. As CoVs replicate their RNA through negative-strand intermediates, nucleoside analogs can get incorporated into RNA intermediates causing chain termination and interfering with polymerase activity. For example, ribavirin, and mizoribine have been studied for their anti-CoV activity, but have only marginal effects (Warren et al., 2014). BCX4430, an adenosine analog, inhibits the polymerase activity in a wide range of RNA viruses, including CoVs (Warren et al., 2014). Micromolar concentrations of acyclic fleximer nucleoside analogs inhibited MERS-CoV and HCoV-NL63 *in vitro* (Peters et al., 2015). Viral helicases catalyze the unwinding of dsRNA intermediates in an ATP-dependent manner. Furthermore, *in vitro* studies have demonstrated the inhibitory effect of bananins and 5-hydroxychromone derivates on the unwinding and atpase activities of SARS-CoV helicase (Tanner et al., 2005; Kim et al., 2011). SSYA10–001 is a triazole that only inhibits the unwinding helicase activity of many CoVs (Kim et al., 2011). Anti-spike antibodies also have been studied *in vivo*; briefly, they bind to the ACE-2 receptor, thus preventing interaction with S protein. The S2 domain of S protein has two heptads repeating regions, HR1 and HR2, which are needed to associate into a 6-helix bundle to mediate membrane fusion. Synthetic peptides that bind to HR1 and HR2 have shown to inhibit SARS-CoV and HCoV-NL63 replication *in vitro*. Exogenous INFs have been used as antiviral agents for animal CoVs and their efficacy has been reported against HCoV-229E. A synergistic effect has been observed to IFN-β and ribavirin against SARs-CoV. In addition, antibiotics, such as actinomycin D were able to inhibit HCoV-229E replication (Kennedy and Johnson-Lussenburg, 1979), while eremomycin, vancomycin, and valinomycin D also have anti-SARS-CoV effects (Adedeji et al., 2014). However, so far, none of these drugs have been approved for COVID-19 treatment.

### Medicinal Plants as Anti-Coronavirus Agents

For over a hundred years, medicinal plants and natural products have played an important role as novel sources for drug development, including antiviral agents (Lin et al., 2014). In fact, numerous medicinal plant extracts and compounds have shown antiviral effects, *in vitro* or *in vivo*, against a wide range of viruses. Thus, it is not surprising that since the COVID-19, SARS, and MERS outbreaks, many researchers have focused on natural products activity, particularly TCM, for both prevention and treatment of novel CoVs infections. In addition, along with their antiviral activities, many natural products are known to increase the immune function, acts as anti-inflammatory and antioxidant agents, at same time that contributes to a balanced healthy status, among other effects.

Natural products, in general, and medicinal plants, in particular, have been widely used globally against CoV infections, with some of these medicinal plant extracts and compounds showing experimental effectiveness in inhibiting CoV growth. For example, it is widely known that the S protein of SARS-CoV-2 commonly interacts with lectin-like receptors in host cells. Thus, researchers have been testing plant-derived lectins for their ability to inhibit the interaction between viral S protein and host receptors (Keyaerts et al., 2007). Lectins derived from Common snowdrop, *Amaryllis*, and Leek revealed to be able to inhibit SARS-CoV replication (Keyaerts et al., 2007). Moreover, specific plant-derived metabolites have also been explored for the development of anti-CoV agents. For example, glycyrrhizin, a bioactive substance extracted from licorice root, is an approved intravenous drug with anti-SARS-CoV activity, although an exact mechanism of action is not entirely clear. Similarly, baicalin derived from *Scutellaria baicalensis*, escin from horse chestnut and reserpine from members of the *Rauwolfia* genus have also revealed promissory anti-SARS-CoV activity (Therapeutic Options for COVID-19 Patients | CDC,; Kim et al., 2008). Traditional Chinese Medicines have been widely used in China and are reported to be effective in SARS-CoV-2 infected individuals (interestingly, in around 90% of Chinese patients), with excellent outcomes (Kim et al., 2008).

While many medicinal plant extracts have been investigated, only eight medicinal plants have demonstrated good inhibitory effects against CoV (Table 2). An aqueous extract of *Houttuynia cordata* Thunb. inhibited the activity of SARS-CoV 3C-like protease (3CLpro) and RNA-dependent RNA polymerase (RdRp) to 50% of control at 1,000 g/mL^107^.

**TABLE 2:** Effects of medicinal herbal extracts on coronavirus.

Plant species	Extraction solvent	Anti-CoV	Target	Ref
*Actaea racemosa* [*syn:Cimicifuga racemosa*]	Methanol	0.0044 ± 0.0029 4.7 ± 1.2 12.2 ± 3.6	Mouse hepatitis virus A59 (MHV-A59) porcine epidemic diarrhea virus (PEDV) vesicular stomatitis virus (VSV)	106
*Houttuynia cordata*	Water	Inhibit the activity of SARS-CoV 3CLpro to 50% of control at the highest testing dose (1,000 g/ml)	SARS-CoV 3C-like protease (3CLpro) and RNA-dependent RNA polymerase (RdRp)	107
*Melia azedarach*	Methanol	0.0198 ± 0.0195 6.7 ± 0.4 20.5 ± 10.5	MHV-A59 PEDV VSV	106
*Coptidis chinensis*	Methanol	<0.0000 5.1 ± 1.5 29.9 ± 24.4	MHV-A59	106
			PEDV	
			VSV	
*Phellodendron amurense*	Methanol	0.0024 ± 0.0012	MHV-A59	106
		5.9 ± 0.4	PEDV	
		40.0 ± 3.8	VSV	
*Paeonia suffruticosa*	Methanol	19.5 ± 3.5	MHV-A59	106
		79.2 ± 0.9	PEDV	
		64.5 ± 15.0	VSV	
*Sophora subprostrata*	Methanol	4.9 ± 2.2	MHV-A59	106
		7.8 ± 0.6	PEDV	
		10.8 ± 7.2	VSV	
*Torreya nucifera*	Ethanol	62% at 100 μg/ml	SARS 3C-like protease (3CLpro)	108

### Natural Products for the Research and Development of Anti-CoV Agents

Investigations of TCM products for antiviral purposes began around 2003, when the SARS CoV first appeared in China in 2002. TCM herbs used for COVID-19 prevention include: *Atractylodes macrocephala, Glycyrrhiza uralensis*, *Astragalus membranaceus*, *Saposhnikoviae divaricate*, *Forsythia suspensa*, *Platycodon grandiflorum*, *Lonicera japonica*, *Atractylodes chinensis*, *Agastache rugosa*, *Cyrtomium fortunei*, *Scrophularia ningpoensis*, *Eupatorium fortunei*, *Ophiopogon japonicus*, *Phragmites communis*, *Dendrobium nobile*, and *Adenophora stricta*, and are the most commonly used herbs in different regions of China (Luo et al., 2020; Yang et al., 2020). For example, in one study, participants who received Yupinfeng powder, consisting of *A. membranaceous*, *Glycyrrhiza glabra*, *S*. *divaricate*, *A. macrocephala*, *L. japonica*, and *F. suspensa*, did not get infected by SARS-CoV (Luo et al., 2020; Yang et al., 2020). In another clinical trial, it was observed that participants who take Kangdu bu fei decoction, composed of *A. membranaceous*, *Aster tataricus*, *Morus alba*, Hedyotic diffusa, *Duchesnea indica* and Scutellaria barbata extracts did not get infected by SARS.110.

Over the years, the anti-CoV activities of naturally occurring compounds derived from commonly used herbal extracts in TCM have been characterized. Briefly, kaempferol derivatives are able to inhibit the 3a ion channel of SARS-CoV (Schwarz et al., 2014). Tetra-O-galloyl-β-d-glucose and luteolin obtained from *Galla chinensis* and *Veronica lina riifolia*, respectively, bind to the surface spike protein of SARS-CoV (Yi et al., 2004). Quercetin and TSL-1 from *Toona sinensis* also inhibited the cell entry of SARS-CoV (Chen et al., 2008). Wang et al. (Wang et al., 2007), used MD simulations and discovered that MOL376, a compound derived from a Chinese medicinal plant inhibited cathepsin L, a target for SARS treatment and suggesting that this compound may be developed as an effective SARS therapy (Wang et al., 2007). In addition, to *Nigella sativa* potent anti-SARS-CoV effects have been reported (Idrees et al., 2020). Other researchers also found that the phytochemical bonducellpin D exhibited broad-spectrum inhibitory effects on SARS-CoV M^pro^ enzyme through an *in silico* approach (Gurung et al., 2020). Similarly, there have been several reports highlighting that glycyrrhizin, an active component isolated from licorice, reduced the replication of two clinical strains of SARS (Cinatl et al., 2003; Hoever et al., 2005). Later in 2005, the same group of researchers described that the semi-synthesis of 15 glycyrrhizic acid (GA) derivatives increased the antiviral activities of such substances against SARS (Cinatl et al., 2003; Hoever et al., 2005). The addition of a 2-acetamido-β-D-glucopyranosylamine to the GA glucoside increased the antiviral activity by 10-fold when compared with GA alone. Furthermore, the addition of an amide or a free 30-COOH function to GA, increased the antiviral activity by ∼70-fold (Hoever et al., 2005), suggesting that GA may be a good starting point for the development of novel anti-CoV drugs.

**FIGURE 2:**
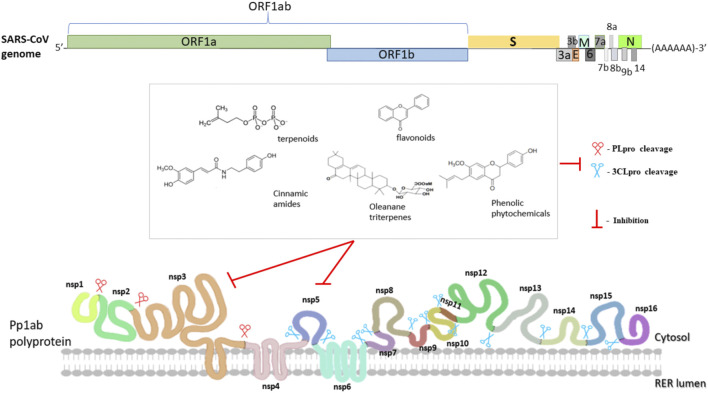
Schematic diagram of SARS-CoV-2 virus genome organization and viral proteases inhibition by natural compounds. The complete genome sequence is ∼30 kb long. Two viral proteases (PLPro and 3CLPro) are responsible for cleaving polyproteins into the functional individual Nsps such as helicase, RDRP and methylases. Nsp3 also known as PLPro is a papain like protease that cleaves Nsp1/Nsp2, Nsp2/Nsp3 and Nsp3/Nsp4 boundaries whereas Nsp5 also known as Mpro or 3CLPro is a serine like protease catalyzing the rest 11 cleavage reactions. Table 3 shows the natural compounds that are known to inhibit these viral proteases.

During the same period, reports of the anti-SARS effects of TCM and isolated substances also started to appear in the literature. The activity and mode of action of some of these bioactive molecules with anti-SARS effects is briefly presented in Figure 2, and discussed below.

In 2004, Wu *et al.*, reported that extracts of *Eucalyptus* spp.*, Lonicera japonica*, and the purified compound ginsenoside-Rb1 (isolated from *Panax ginseng*) reduced the SARS-CoV replication *in vitro*, although only at high concentrations (100 μM) (Wu et al., 2004). Other TCM preparations, composed of *artemisia annua, Lycoris radiata, Pyrrosia lingua*, and *Lindera aggregata* extracts also reduced the *in vitro* replication of SARS-CoV (Li et al., 2005). These four plant extracts inhibited viral replication in Vero cell-based assays, with median effective concentrations (EC_50_) ranging from 2.4 to 88.2 μg/ml, with *L. radiata* extract being the most active. Interestingly, bioassay-guided fractionation of *L. radiata* extract led to the isolation and identification of lycorine as the most active anti-SARS-CoV agent, with EC_50_ concentrations for lycorine ranging from 15.7 to 48.8 nM (Li et al., 2005). Similarly, in 2005, Lin et al. reported that extracts and phenolic compounds from *Isatis indigotica* root inhibited the SARS chymotrypsin CoV 3C-like protease (3CL^pro^) (Lin et al., 2005). 3CL^pro^ is the viral protease responsible for proteolytic processing of replicase polyproteins in CoVs, necessary for viral life cycle (Lin et al., 2005). Therefore, this protease is considered to be an important molecular target for the development of new anti-CoV drugs.

When screening 720 naturally occurring substances, Chen et al. found that compounds from *Camellia sinensis* (tea leaves) inhibited the 3CL^Pro^ activities (Inhibition of SARS-CoV 3C-like Protease Activity by Theaflavin-3,3’-digallate (TF3)). Both tannic acid and 3-isotheaflavin-3-gallate had a median inhibitory concentration in the range of 3–7 µM. Furthermore, screening of tea extracts showed that extracts from green, oolong, Puer, and black teas were also active, with Puer and black teas being the most active *in vitro*. Another tea substance with 3CL^pro^ inhibitory activity was identified as theaflavin-3,3′-digallate (Inhibition of SARS-CoV 3C-like Protease Activity by Theaflavin-3,3’-digallate (TF3)). In 2006, Cheng et al. reported that specific triterpene glycosides isolated from *Bupleurum*, *Hetermorpha* and *Scrophularia* species, namely saikosaponins A, B2, C and D, inhibited the human HCoV-229E replication *in vitro* (Cheng et al., 2006). These compounds also reduced early stage HCoV-22E infections, meaning that they may have possible preventative effects by suppressing both attachment and penetration of CoV into cells (Cheng et al., 2006). Ho *et al,* also tested three TCM-derived herbal extracts from roots of *Rheum officinale*, and roots and vine of *Polygonum multiflorum*, and obtained median inhibitory concentrations ranging from 1 to 10 μg/ml (Ho et al., 2007). Mechanistically, the extracts inhibited the SARS-CoV S protein binding. Moreover, using bioassay-guided fractionation, the authors isolated the anthraquinone compound emodin from *Rheum* and *Polygonum* genus, with the observed effects being attributed its presence in the extracts; briefly, this compound inhibited the virus replication by inhibition of the interaction between CoV S protein and hACE2 receptor (Schwarz et al., 2011). Emodin was also shown to inhibit the 3a protein ion channel, which forms a cation-selective channel that allows the viral release into the infected cells. Thus, taken together, substances that can inhibit the 3a protein and the virus release are also interesting for the development of anti-SARS agents (Ho et al., 2007; Schwarz et al., 2011).

In another study, Wen et al., investigated the anti-SARS-CoV activity of 221 natural compounds (Wen et al., 2007). As main outcomes, the authors demonstrated that many of the compounds reduced virus replication, particularly eight abietane-type diterpenes, two labdane-type diterpenes, two sesquiterpenes, and two triterpenes. Also, four lignan compounds (including savinin, honokiol, and magnolol), as well as curcumin, strongly inhibited SARS-CoV activity at concentrations of 3.3–20 μM (Wen et al., 2007). α-cadinol and hinokinin also significantly inhibited viral replication at 1 μM, suggesting that abietane-type diterpenoids and lignans may be interesting sources for the development of novel anti-SARS CoV agents. Kim et al. (Kim et al., 2008), investigated the effects of 22 Chinese herbs extracts on the replication of two CoVs, namely mouse hepatitis virus A59 (MHV-A59) and porcine epidemic diarrhea virus (PEDV). Among the studied extracts, *C. racemosa*, *Melia azedarach*, *Coptidis chinensis*, *P. amurense*, and *S. subprostrata* reduced the MHV-A59 replication with an EC_50_ ranging from 2.0 to 27.5 μg/ml, with both RNA and protein expression being also changed (Kim et al., 2008). In 2008, Lau et al., investigated the antiviral and immune effects of *Houttuynia cordata*, a TCM plant used to treat pneumonia (Lau et al., 2008). The aqueous extract increased mouse spleen lymphocytes proliferation *in vitro*, and increased IL-2 and IL-10 production. Treatment with HC aqueous extract also increased the proportion of CD4^+^ and CD8^+^ T cells, besides to be able to inhibit the SARS-CoV 3CL^pro^ and suppress RNA-dependent RNA polymerase activity (Lau et al., 2008). Zhuang et al., investigated the anti-SARS activities of a butanol fraction of Cinnamon bark, and its isolated chemical constituents. The butanol fraction of *Cinnamomum verum* weakly inhibited the replication of wild-type SARS-CoV and HIV/SARS-CoV S pseudovirus, with an IC_50_ of 7.8–43.1 and 149.5–283.4 μg/ml, respectively (Zhuang et al., 2009). Two substances, namely procyanidin A2 and B1, exerted anti-SARS-CoV activity with median inhibitory concentrations of 29.9–41.3 μM and 15.69–37.35 μM, respectively (Zhuang et al., 2009). Around the same time, several Chinese patents were filed, showing that naturally occurring diterpenes were able to co-crystallize with 3CL^pro^ of the SARS virus (Rao et al., 2007; Method for separating SARS coronavirus main proteinase inhibitor from traditional Chinese medicine, 2009; Method for screening SARS corona virus major protease inhibitor from traditional Chinese medicine and screened SARS corona virus major protease inhibitor, 2010; Kumar et al., 2013). In this report, pseurata A, B and C, leukamenin E, glaucocalyxin B and D, liangshanin A, all inhibited 3CL^pro^, meaning that all may be considered potential candidates for new anti-SARS CoV agents formulation. Moreover, other naturally occurring compounds, such as scutellarein, quercetagetin, myricetin, and robinetin were reported to inhibit SARS 3CL^pro^ (IC_50_ = 25 μM) (Song et al., 2009).

In 2010, Kim et al., assessed 19 herbal extracts used in TCM and assessed their effects against CoV MHV-A59. Among the studied extracts, that derived from *Sanguisorba officinalis*, *Acanthopanax gracilistylus* and *Torilis japonica* markedly reduced the MHV-A59 replication, as well as the viral RNA and protein levels (Song et al., 2009). These extracts were also able to reduce viral replication in the JHM strain of MHV, porcine epidemic diarrhea virus, and vesicular stomatitis virus. Median effective concentrations of such extracts were in the range of 0.8–3.7 μg/ml. *A. gracilistylus* and *T. japonica* extracts also displayed anti-inflammatory activities and reduced COX-2 activity in MHV-A59-infected cells. They also activated the extracellular signal-related kinase (ERK) and p38 or ERK alone (Kim et al., 2010). Moreover, in 2010, Rhy et al. (Ryu et al., 2010), assessed the anti-SARS-CoV effects of four quinone-methide triterpenes, celastrol, pristimerin, tingenone and iguesterin, isolated and identified from *Triterygium regelii*. These triterpenes inhibited the SARS-CoV 3CL^pro^ activity with median inhibitory concentrations of 10.3, 5.5, 9.9, and 2.6 μM, respectively (Ryu et al., 2010). In 2011, a *Celastrus orbiculatus*-derived extract and its fractions inhibited the 3CL^pro^ activity with an IC_50_ of 17.8–38.9 μg/ml (Kumar et al., 2013). An ethanol extract and ethyl acetate fraction of brown seaweed line tree inhibited the SARS CoV with an IC_50_ of 14.7 and 8.5 μg/ml, respectively (Kumar et al., 2013). The isolated active substances revealed significant anti-CoV effects, with celastrol, pristimerin, tingenone and iguesterin having IC_50_ values ranging from 2.6 to 10.3 nM. Other natural compounds that have been identified as inhibitors of SARS-CoV helicase nsP13 and 3CL^pro^, include the phenolic compounds myricetin and scutellarein isolated from *Torreya nucifera* (Ryu et al., 2010; Yu et al., 2012). Wen et al., in 2011, screened over 200 Chinese herbal extracts for their anti-SARS-CoV activities in cultured Vero E6 cells. As main findings, the authors stated that six herbal extracts, namely from *Gentiana scabra*, *Dioscorea batatas*, *Cassia tora* and *Taxillus chinensis* and two *Cibotium barometz* extracts inhibited the SARS-CoV replication at concentrations between 25 and 200 μg/ml (Wen et al., 2011). Moreover, *D. batatas* and *C. barometz* methanol extracts significantly inhibited the SARS-CoV 3CL^pro^ activity, with a median inhibitory concentration of 39–44 μg/ml, respectively (Wen et al., 2011). Also, Chang et al. reported the anti-human CoV activity of *Euphorbia neriifolia* and its derived triterpenes, with 3β-friedelanol revealing to be more effective than the control actinomycin D, thus suggesting the importance of the friedelane skeleton as a basis for drug development (Chang et al., 2012).

More recently, rocaglamide and silvestrol, two naturally occurring compounds that were isolated from *Aglaia* species, have also proved to have excellent antiviral activity (Schulz et al., 2020). Furthermore, a recent study by Muller et al. (Müller et al., 2018), reported that silvestrol, significantly inhibited cap-dependent viral mRNA translation in CoV-infected human embryonic lung fibroblast cells. The median effective concentrations were of 1.3–3 nM, with significant effects being stated both on MERS-CoV and HCoV-229E. The activity of silvestrol was also investigated in the highly pathogenic MERS-CoV, using peripheral blood mononuclear cells. Mechanistically, silvestrol inhibited the CoV structural and nonstructural protein (N, nsp8) expression, as well as viral replication and transcription complexes formation (Song et al., 2009). Furthermore, *Stephania tetrandra* extracts and isolated alkaloids, tetrandrine (TET), fangchinoline (FAN), and cepharanthine (CEP) were investigated in HCoV-OC43-infected MRC-5 human lung cells. These substances significantly inhibited the virus replication and increased cell death with an IC_50_ range of 0.33–1.01 μM. Moreover, TET, FAN, and CEP inhibited the S and N proteins expression (Kim et al., 2019).

### Traditional Chinese Herbal Medicine and Western Medicine: Clinical Approach

Chinese herbal medicines used in combination with Western medicines (antibiotics and corticosteroids) vs. Western medicines alone have been increasingly assessed for their safety and efficacy, and its impact in patients infected with SARS-CoV is not an exception (Liu et al., 2012; Wang et al., 2020).

A systematic review identified a quasi-randomized controlled trial (RCT) and 22 RCTs of Chinese herbal medicines in combination with Western medicines for the treatment of SARS-CoV that met the inclusion criteria (Liu et al., 2012). However, 10 RCTs were not included in the final analyses due to lack of outcome measures and randomization. Twelve RCTs and one quasi-RCT were finally evaluated involving a total of 640 SARS-CoV-infected patients. A total of 12 Chinese herbal combinations were taken into consideration. The primary outcome was mortality, while secondary outcomes included symptoms’ reduction, pulmonary infiltration, quality of life, duration of hospitalization, corticosteroids’ use, and occurrence of adverse events. Data obtained in this systematic review suggested that the combination of Chinese herbal medicines with Western medicines were not more effective than Western medicines alone in reducing mortality (Liu et al., 2012). However, the combination of both medicines was effective for reducing SARS-CoV symptoms, including fever, cough, breathing difficulties, and serious sequelae, when compared with Western medicines alone. Also, both medicines, used in combination seemed to be effective in reducing lung infiltration and the corticosteroids use, and in improving the quality of life of SARS-CoV-infected patients, although did not reduce the number of days in the hospital. All these Chinese herbal treatments were combination prescriptions, that have been personalized for patients. Also worth of note is that a recently published study revealed that four COVID-19 patients receiving treatment with Shufeng Jiedu Capsule, a TCM, in combination with lopinavir/ritonavir and arbidol had an improvement in respiratory symptoms (Wang et al., 2020).

Thus, the combination of TCM with Western medicine in SARS-CoV-infected patients seems to be promising. However, despite the recent advances, more in-depth clinical trials are needed toward a clearer understanding on the safety and effectiveness of Chinese herbal medicines used in combination with Western drugs, as well as of detailed studies reporting the occurrence of adverse events.

## Anti-Coronavirus Effects of Naturally Occurring Bioactive Compounds

Many natural products have been reported to inhibit CoV growth or the activity of targeted enzymes, such as SARS-CoV PLpro, with the most promissory ones belonging to the terpenes and flavonoids classes (Hamill et al., 2006; Park et al., 2012; Cho et al., 2013; Kim et al., 2014; Yang et al., 2015; Jo et al., 2019; Jo et al., 2020). Most of tested natural products have inhibitory activity on CoV (Table 3) with IC_50_ values ranging from 0.86 to 283.5 µM on SARS-Cov 3CLPro and PLPro and from 1.7 to 19.9 µM on hCoV 229E. Scutellarein, hirtutenone, cryptotanshinone, myrcetin, rosmariquinone and tanshinone IIA have shown the highest inhibitory effect with IC_50_ values below 5 µM (Park et al., 2012; Cho et al., 2013; Yang et al., 2015).

**TABLE 3:** Effects of natural products on coronavirus.

Compound	Source	Anti-Cov	Target/Strains	Ref
18-Hydroxyferruginol	*Tripterygium regelii*	220.8 ± 10.4	SARS 3C-Like protease (3clpro)	^Ryu et al. (2010)^
18-Oxofer- ruginol	*Tripterygium regelii*	163.2 ± 13.8	SARS 3C-Like protease (3clpro)	^Ryu et al. (2010)^
30-O-Methyldiplacol	*Paulownia tomentosa*	9.5 ± 0.10	SARS-Cov Plpro	^Cho et al. (2013)^
30-O-Methyldiplacone	*Paulownia tomentosa*	13.2 ± 0.14	SARS-Cov Plpro	^Cho et al. (2013)^
4′-O-Methylbavachalcone	*Psoralea corylifolia*	10.1 ± 1.2	SARS-Cov) papain-like protease (Plpro)	^Kim et al. (2014)^
40-O-Methyldiplacol	*Paulownia tomentosa*	9.2 ± 0.13	SARS-Cov Plpro	^Cho et al. (2013)^
40-O-Methyldiplacone	*Paulownia tomentosa*	12.7 ± 0.19	SARS-Cov Plpro	^Cho et al. (2013)^
6-Geranyl-40,5,7-Trihydroxy-30,50-Dimethoxyflavanone	*Paulownia tomentosa*	13.9 ± 0.18	SARS-Cov Plpro	^Cho et al. (2013)^
Abietic acid	*Pinus* spp.	189.1 ± 15.5	SARS 3C-Like protease (3clpro)	^Ryu et al. (2010)^
Amentoflavone	*Tripterygium regelii*	8.3 ± 1.2	SARS 3C-Like protease (3clpro)	^Ryu et al. (2010)^
Apigenin	—	280.8 ± 21.4	SARS 3C-Like protease (3clpro)	^Ryu et al. (2010)^
Bavachinin	*Psoralea corylifolia*	38.4 ± 2.4	SARS-Cov) papain-like protease (Plpro)	^Kim et al. (2014)^
Bilobetin	*Tripterygium regelii*	72.3 ± 4.5	SARS 3C-Like protease (3clpro)	^Ryu et al. (2010)^
Corylifol A	*Psoralea corylifolia*	32.3 ± 3.2	SARS-Cov) papain-like protease (Plpro)	^Kim et al. (2014)^
Cryptotanshinone	*Salvia miltiorrhiza*	226.7 ± 6.2; 0.8 ± 0.2	SARS-Cov 3CL (Pro) and PL (Pro)	^Park et al. (2012)^
Curcumin	*Curcuma longa*	5.7 ± 0.3	SARS-Cov Plpro	^Yang et al. (2015)^
Dihydrotanshinone I	*Salvia miltiorrhiza*	38.7 ± 8.2; 8.8 ± 0.4	SARS-Cov 3CL (Pro) and PL (Pro)	^Park et al. (2012)^
Diplacone	*Paulownia tomentosa*	10.4 ± 0.16	SARS-Cov Plpro	^Cho et al. (2013)^
Esculetin-4-Carboxylic acid ethyl Ester	*Axinella corrugata*	46	SARS-Cov 3clpro	^Hamill et al. (2006)^
Ferruginol	*Tripterygium regelii*	49.6 ± 1.5	SARS 3C-Like protease (3clpro)	^Ryu et al. (2010)^
Ginkgetin	*Tripterygium regelii*	32.0 ± 1.7	SARS 3C-Like protease (3clpro)	^Ryu et al. (2010)^
Helichrysetin	*Helichrysum odaratissimum*	67.04 µm	MERS-Cov 3C-Like protease (3clpro)	^Jo et al. (2019)^
Herbacetin	—	33.17	SARS 3C-Like protease (3clpro)	^Jo et al. (2020)^
Herbacetin	—	40.59	MERS-Cov 3C-Like protease (3clpro)	^Jo et al. (2019)^
Hinokiol	*Tripterygium regelii*	233.4 ± 22.2	SARS 3C-Like protease (3clpro)	^Ryu et al. (2010)^
Hirsutanonol	*Alnus japonica*	7.8 ± 1.7	SARS-Cov Plpro	^Yang et al. (2015)^
Hirsutenone	*Alnus japonica*	4.1 ± 0.3	SARS-Cov Plpro	^Yang et al. (2015)^
Isobavachalcone	*Psoralea corylifolia*	35.85	MERS-Cov 3C-Like protease (3clpro)	^Jo et al. (2019)^
Isobavachalcone	*Psoralea corylifolia*	7.3 ± 0.8	SARS-Cov) papain-like protease (Plpro)	^Kim et al. (2014)^
Isopimaric acid	*Tripterygium regelii*	283.5 ± 18.4	SARS 3C-Like protease (3clpro)	^Ryu et al. (2010)^
Kayadiol	*Tripterygium regelii*	137.7 ± 12.5	SARS 3C-Like protease (3clpro)	^Ryu et al. (2010)^
Luteolin	*Reseda luteola*	20.0 ± 2.2	SARS 3C-Like protease (3clpro)	^Ryu et al. (2010)^
Methyl Dehydroabi- Etate	*Tripterygium regelii*	207.0 ± 14.3	SARS 3C-Like protease (3clpro)	^Ryu et al. (2010)^
Methyl Tanshinonate	*Salvia miltiorrhiza*	21.1 ± 0.8; 9.2 ± 2.8	SARS-Cov 3CL (Pro) and PL (Pro)	^Park et al. (2012)^
Mimulone	*Paulownia tomentosa*	14.4 ± 0.27	SARS-Cov Plpro	^Cho et al. (2013)^
Myricetin	—	2.71 ± 0.19	—	^Yu et al. (2012)^
Neobavaisoflavone	*Psoralea corylifolia*	18.3 ± 1.1	SARS-Cov) papain-like protease (Plpro)	^Kim et al. (2014)^
N-Trans-Caffeoyltyramine	*Tribulus terrestris*	44.4 ± 0.6	SARS-Cov Plpro	^Song et al. (2014)^
N-Trans-Coumaroyltyramine	*Tribulus terrestris*	38.8 ± 0.4	SARS-Cov Plpro	^Song et al. (2014)^
N-Trans-Feruloyloctopamine	*Tribulus terrestris*	26.6 ± 0.5	SARS-Cov Plpro	^Song et al. (2014)^
N-Trans-Feruloyltyramine	*Tribulus terrestris*	70.1 ± 0.7	SARS-Cov Plpro	^Song et al. (2014)^
O-acetyl-18-Hydroxyferruginol	*Tripterygium regelii*	128.9 ± 25.2	SARS 3C-Like protease (3clpro)	^Ryu et al. (2010)^
Oregonin	*Alnus japonica*	20.1 ± 2.2	SARS-Cov Plpro	^Yang et al. (2015)^
Pectolinarin	*Cirsium* spp.	37.78	SARS 3C-Like protease (3clpro)	^Jo et al. (2020)^
Platyphyllone	*Alnus japonica*	>200	SARS-Cov Plpro	^Yang et al. (2015)^
Platyphyllonol-5-Xylo- Pyranoside	*Alnus japonica*	>200	SARS-Cov Plpro	^Yang et al. (2015)^
Psoralidin	*Psoralea corylifolia*	4.2 ± 1.0	SARS-Cov) papain-like protease (Plpro)	^Kim et al. (2014)^
Quercetin	*Toona sinensis*	23.8 ± 1.9	SARS 3C-Like protease (3clpro)	^Ryu et al. (2010)^
Quercetin 3-Β-D-Glucoside	—	37.03	MERS-Cov 3C-Like protease (3clpro)	^Jo et al. (2019)^
Rhoifolin	*Rhus saccedanea*	27.45	SARS 3C-Like protease (3clpro)	^Jo et al. (2020)^
Rosmariquinone	*Salvia miltiorrhiza*	14.4 ± 0.7; 4.9 ± 1.2	SARS-Cov 3CL (Pro) and PL (Pro) assay	^Park et al. (2012)^
Rubranol	*Alnus japonica*	12.3 ± 0.9	SARS-Cov Plpro	^Yang et al. (2015)^
Rubranoside A	*Alnus japonica*	9.1 ± 1.0	SARS-Cov Plpro	^Yang et al. (2015)^
Rubranoside B	*Alnus japonica*	8.0 ± 0.2	SARS-Cov Plpro	^Yang et al. (2015)^
Saikosaponin A	*Bupleurum* spp.*, heteromorpha* spp. *And scrophularia scorodonia*	8.6 ± 0.3	Human Coronavirus 229E	^Cheng et al. (2006)^
Saikosaponin B2	*Bupleurum* spp.*, heteromorpha* spp. *And scrophularia scorodonia*	1.7 ± 0.1	Human Coronavirus 229E	^Cheng et al. (2006)^
Saikosaponin C	*Bupleurum* spp.*, heteromorpha* spp. *And scrophularia scorodonia*	19.9 ± 0.1	Human Coronavirus 229E	^Cheng et al. (2006)^
Saikosaponin D	*Bupleurum* spp.*, heteromorpha* spp. *And scrophularia scorodonia*	13.2 ± 0.3	Human Coronavirus 229E	^Cheng et al. (2006)^
Sciadopitysin	*Tripterygium regelii*	38.4 ± 0.2	SARS 3C-Like protease (3clpro)	^Ryu et al. (2010)^
Scutellarein	*Scutellaria* spp.	0.86 ± 0.48	Colorimetry-based ATP Hydrolysis	^Yu et al. (2012)^
Tanshinone	*Salvia miltiorrhiza*	38.7 ± 8.2; 8.8 ± 0.4	SARS-Cov 3CL (Pro) and PL (Pro)	^Park et al. (2012)^
Tanshinone IIA	*Salvia miltiorrhiza*	89.1 ± 5.2; 1.6 ± 0.5	SARS-Cov 3CL (Pro) and PL (Pro)	^Park et al. (2012)^
Tanshinone IIB	*Salvia miltiorrhiza*	24.8 ± 0.8; 10.7 ± 1.7	SARS-Cov 3CL (Pro) and PL (Pro)	^Park et al. (2012)^
Terrestriamide	*Tribulus terrestris*	21.5 ± 0.5	SARS-Cov Plpro	^Song et al. (2014)^
Terrestrimine	*Tribulus terrestris*	15.8 ± 0.6	SARS-Cov Plpro	^Song et al. (2014)^
Tomentin A	*Paulownia tomentosa*	6.2 ± 0.04	SARS-Cov Plpro	^Cho et al. (2013)^
Tomentin B	*Paulownia tomentosa*	6.1 ± 0.02	SARS-Cov Plpro	^Cho et al. (2013)^
Tomentin C	*Paulownia tomentosa*	11.6 ± 0.13	SARS-Cov Plpro	^Cho et al. (2013)^
Tomentin D	*Paulownia tomentosa*	12.5 ± 0.22	SARS-Cov Plpro	^Cho et al. (2013)^
Tomentin E	*Paulownia tomentosa*	5.0 ± 0.06	SARS-Cov Plpro	^Cho et al. (2013)^

However, in general, although medicinal plants and natural products have anti-CoV activity, most of the results are still preliminary. Only a few studies assessed the effectiveness of natural products directly on the virus because most of them targeted CoV proteases. Therefore, the reported effects of these substances on the whole virus should be confirmed and then experiments should be performed on animal models of CoV infections before starting clinical trials.

### Paving the Way for Clinical Applications Against SARS-CoV-2 in Humans

The emergence of SARS-CoV-2 as a cause of the COVID-19 worldwide pandemic has prompted an urgent need to research and develop new vaccines and drugs to tackle its pathogenesis. Even with the advent of effective vaccines for SARS-CoV-2, there are currently few direct acting antiviral and other drugs to treat the symptoms of COVID-19. There is an urgent need for the development of such therapies as vaccines are not 100% effective and some patients may still have symptoms, albeit less severe, the vaccines may require annual shots, and a high percentage of the global population need to be vaccinated before the vaccines can offer maximum protection.

Natural products, with their diversity of chemical classes and structures, and preliminary data suggesting that some of these compounds may be active against CoVs offer an excellent starting point for drug discovery in this field. Fortunately, researchers have been able to identify and sequence proteins that are crucial elements for viral infection, replication, and virus-host interactions that can be used as tools for screening novel antiviral substances, including natural products. In fact, preliminary data suggest that naturally occurring bioactive compounds that can target: 1) SARS-CoV-2 entry/fusion; 2) virus uncoating inside the host cell, 3) nucleoside and non-nucleoside reverse transcriptase, 4) viral protease, 5) and viral release. In addition, data suggest that natural products may: 1) block SARS-CoV-2 entry by inhibiting its attachment and fusion to host cells, 2) stop viral replication by inhibiting viral protease and viral nucleic acid as well as protein synthesis, 3) block viral survival in host cells, and 4) boost the host immune response. The effects of natural products on the immune system.

As briefly stated above, SARS-CoV-2 infection activates the host immune cells that cause cytokine storm, and this activation is directly linked to disease severity and poor prognosis (Huang et al., 2020). There are many reports of natural products that have been shown to modulate the host’s immune responses (Khanna et al., 2020; Silveira et al., 2020). Medicinal plant extracts and natural compounds have the potential to be used alone or as adjunct therapies in combination with current antiviral treatments and other drugs. In fact, TCM therapies, have been suggested to be effective in human studies in combination with conventional treatments. Thus, medicinal plant extracts and natural compounds make excellent candidates for testing as novel antiviral, anti-inflammatory and immune enhancing therapies, alone or in combination with other drugs to develop more effective, and safer clinical interventions and promissory outcomes.

## Conclusion and Future Perspectives

Previous CoVs, and the new CoV, named SARS-CoV-2, have markedly compromised the global public health. The approval of recent vaccines has significantly improved the long-term outcomes for COVID-19 infections however, vaccines are not 100% effective and some serious symptoms still persist. In addition, it will take a significant period of time to vaccinate everyone globally, thus there is still a critical need to research and develop safe and effective drug therapies.

Natural products have been successfully used for centuries for treating a plethora of diseases, including infectious diseases. To date, the published data suggest that natural products make excellent candidates to serve as a starting point for the search and development of novel treatments and preventative agents for CoVs infections. However, so far, much of this research is *in vitro*, and few experiments have been performed in animal models. Furthermore, new randomized clinical trials need to be performed, as some of the trials of TCMs have shown a reduction in both symptoms and sequelae, but not in mortality rates (Huang et al., 2020). Moreover, the application of new methods/technologies, such as high throughput screening and molecular biology must also be employed to isolate bioactive compounds from plant extracts. Thus, future studies should focus on these aspects of natural products research to more effectively research and develop these compounds as effective treatment strategies for SARS-CoV-2 infection. Taken together, data discussed here highlight the need for a concerted global effort to test natural products for their ability to fight the emerging CoVs strains, particularly in combination with conventional drugs toward to provide more effective, safer and targeted therapies.

## Abbreviations

3CLpro, chymotrypsin 3C-like protease; BCoV, bovine coronavirus; CCV, canine coronavirus; CEP, cepharanthine; COVID-19, coronavirus disease; COX-2, cyclooxygenase-2; CoV, Coronavirus; DPP4, Dipeptidyl-peptidase-4; ERK, extracellular-signal regulated kinase; FAN, fangchinoline; FCoV, feline coronavirus; IBV, infectious bronchitis virus; JAK-STAT, Janus kinase-signal transducer and activator of transcription; MDA5, Melanoma differentiation associated protein-5; MERS CoV, Middle East respiratory syndrome coronavirus; MHV, Murine hepatitis virus; NF-κB, Nuclear factor-κB; PEDV, porcine epidemic diarrhea virus; PLpro, Papain like protease; PRCoV, Porcine respiratory coronavirus; RDRP, RNA dependent RNA polymerase; SARS, severe acute respiratory syndrome; SARS-CoV-2, severe acute respiratory syndrome coronavirus-2; SARS-CoV, severe acute respiratory syndrome coronavirus; TCM, traditional Chinese medicine; TET, tetrandrine; TGEV, transmissible gastroenteritis virus; TLR, Toll like receptor; UPR, unfolded protein response; hACE-2, human angiotensin converting enzyme-2; hCoVs, human coronaviruses.
